# Multi-Hop Routing-Based Optimization of the Number of Cluster-Heads in Wireless Sensor Networks

**DOI:** 10.3390/s110302875

**Published:** 2011-03-03

**Authors:** Choon Sung Nam, Young Shin Han, Dong Ryeol Shin

**Affiliations:** School of Information and Communication Engineering, Sungkyunkwan University, Suwon 440-774, Korea; E-Mails: namgun99@skku.edu (C.S.N.); yshan95@ewhain.net (Y.S.H.)

**Keywords:** sensor networks, clustering method, multi-hop routing, optimal cluster-heads

## Abstract

Wireless sensor networks require energy-efficient data transmission because the sensor nodes have limited power. A cluster-based routing method is more energy-efficient than a flat routing method as it can only send specific data for user requirements and aggregate similar data by dividing a network into a local cluster. However, previous clustering algorithms have some problems in that the transmission radius of sensor nodes is not realistic and multi-hop based communication is not used both inside and outside local clusters. As energy consumption based on clustering is dependent on the number of clusters, we need to know how many clusters are best. Thus, we propose an optimal number of cluster-heads based on multi-hop routing in wireless sensor networks. We observe that a local cluster made by a cluster-head influences the energy consumption of sensor nodes. We determined an equation for the number of packets to send and relay, and calculated the energy consumption of sensor networks using it. Through the process of calculating the energy consumption, we can obtain the optimal number of cluster-heads in wireless sensor networks.

## 1. Introduction

Wireless sensor networks (WSNs) are organized by wireless nodes for monitoring the existing conditions in a specific area. Sensor nodes consists of three basic devices: a sensor that observes changes in the surroundings, a processor that handles the sensing data, and a wireless transmitter/receiver set that sends processed data to a base station (BS) that collects, analyzes and sends the sensing data to an external network [1]. Generally, the energy efficiency of sensor nodes is one of the most critical issues for sensor networks due to their restricted energy resources and communication range. Additionally, it is difficult to replace or recharge their batteries. Therefore, in order to reduce the energy consumption of sensor nodes, sensor networks must use energy efficiently, as well as use some scalable clustering technique as a method for organizing the networks. A clustering method is a way to divide networks into local clusters which must consider load balancing and energy distribution of sensor nodes in order to prolong network lifetime. Sensor nodes should, using clustering methods, use many-to-one communication for transmitting the sensing data to the cluster-heads or base station and adopt multi-hop communication for packet relay contrary to existing work [2], since nodes with a restricted communication radius cannot directly communicate with nodes outside this radius [3].

The cluster-head is in charge of transmitting sensing data from its own local cluster, as well as collecting and compressing multiple data before sending them to a sink node. They consume more energy than other sensor nodes as a result of these additional tasks. Therefore, it is desirable that all sensor nodes should take on the role of a cluster-head, equally and randomly. Based on the number of cluster-heads, the size of a local cluster may change. It is important to construct adaptive clusters because the number of cluster-heads has an effect on the energy consumption of the cluster-head and the sending of member nodes’ data. As more cluster-heads become available and the smaller the size of a local cluster, the smaller the amount of packets required to be sent will be. With additional cluster-heads, however, there is an increase in the number of packets needed for cluster-heads to communicate to a sink or base station, therefore increasing the energy consumption as a result of clustering. In this paper, we determine the energy variation rate of whole sensor networks based on the energy consumption of a local cluster (intra-cluster) and between local clusters (inter-cluster) using equations. Further, based on this result, we propose an optimal number of cluster-heads in wireless sensor networks based on multi-hop routing.

## 2. Related Works

A typical application that sensor node networks support is the monitoring of some remote environment. Since individual nodes’ data in a sensor network are often correlated, the end user does not require all the redundant data, but rather some high-level fraction of the data that accurately describes the events occurring in the environment. To achieve this, LEACH [4] allows all data from nodes within the cluster to be processed locally, reducing the data set that needs to be transmitted to the end user. For the development of LEACH, there are some assumptions about the sensor nodes and the underlying network model. For sensor nodes, all nodes can transmit with enough power to reach the BS if needed, that the nodes can use power control to vary the amount of transmission power, and that each node has the computational power to support different MAC protocols and perform signal processing functions. For the network, nodes can always have data to send to the end user and nodes located close to each other have correlated data. In the LEACH algorithm, the sensor nodes belong to each local cluster. All member nodes send sensing data to their own cluster head (CH), and the CHs send the aggregated data to the sink node after collecting sensing data from their own member nodes and processing them. In LEACH, the transmission method of nodes is based on single-hop communication. That means that the transmission method between the CH and member nodes of the intra-cluster and between the CHs and a sink node is single-hop communication. This communication method has been used in many clustering algorithms for WSNs. In LCA [5,6] and adaptive clustering [7], the CH can directly connect to all nodes in its cluster like LEACH. CLUBS [8] forms the clusters with a maximum of two hops, regardless of network size. Besides, the other clustering methods which are revised and extended versions of LEACH such as LEACH-C [4], HEED [9], EAP [10] focus on cluster head selection for energy efficiency. The communication range of sensor nodes is based on IEEE 802.15.4 (LR-WPAN) which is one of the transmission standards for WSNs. IEEE 802.15.4 typically extends up to 10 m in all directions [11]. However, the above clustering algorithms did not adopt realistic communication radius and multi-hop communication method in intra- and inter-clusters [12]. After constructing clusters, the CHs in local clusters have the authority to operate data transmission during a communication between member nodes. As a local cluster size and the distance between a CH and a sink might be bigger than the restricted radius, any communication should be based on a multi-hop method and is thus affected by the number of cluster heads. That means that if the CHs are increased, the distance between a CH and member nodes is decreased in intra-clusters and the distance between a CH and a sink is increased in inter-clusters. To increase the distance is the same as increasing the number of required relay packets, so we need to know the optimal number of clusters for energy-efficient communication.

## 3. Optimal Number of Cluster-Heads

This paper is based on the following assumptions: all sensor nodes can communicate with other nodes in a possible communication radius, R [13]. The state of communication can be divided into intra-cluster communication and inter-cluster communication by the role of cluster-heads. Intra-cluster communication is the state of sending a member node’s sensing data to a cluster-head and assumes that a cluster-head sets up their time division multiple access TDMA schedule to allow sufficient time for transmission. Therefore, member nodes can generate and send data by scheduling. Inter-cluster communication is the state of sending data to sink by cluster heads using the TDMA scheduler. The cluster-heads are equally and randomly selected at a constant rate. All sensor nodes use the multi-hop communication method for relay packets because some of the nodes cannot communicate to the sink directly.

### 3.1. Radio Model for Energy Consumption

The radio model for a sensor node’s energy consumption assumes that all of the nodes maintain a minimum level of successful communication. There are different energy calculations between transmitting and receiving data. Generally, a radio model defines the energy consumption of a transmitter-receiver as *E_elec_ J/bit*, and the energy consumption of a transmitter amplifier by a signal to noise ratio as *E_amp_ J/bit*. Therefore, the following equation is required for sending a *k* (bit) message to a node with a distance of *d* (meters) [4]:

(1) $$E_{\mathrm{Tx}}(k,d)=k\;E_{\mathrm{elec}}+{\mathrm{kE}}_{\mathrm{amp}}d^{2}$$

Equation (1), *E_Tx_*, is the transmitting energy of a radio beam:

(2) $$E_{\mathrm{Rx}}(k,d)={\mathrm{kE}}_{\mathrm{elec}}$$

In Equation (2), *E_R_*, is the receiving energy of a radio beam. We set up a distance d to R for multi-hop based clustering as seen in the following energy equations:

(3) $$E_{\mathrm{Rx}}(k,R)={\mathrm{kE}}_{\mathrm{elec}}=l$$

(4) $$E_{\mathrm{Tx}}(k,R)=k\;E_{\mathrm{elec}}+{\mathrm{kE}}_{\mathrm{amp}}R^{2}=l+{\mu R}^{2}$$

### 3.2. Calculation for Clustering

The clustering energy consumption of sensor networks is divided into two categories: intra energy generated in a local cluster and inter energy generated when a cluster-head sends the aggregated data to a sink. In each category, the total energy consumption of the networks, *E_cluster_*, can be measured by adding the cluster-heads energy, *E_ch_*, to the member nodes energy, *E_member_* as follows:

(5) $$E_{\mathrm{Tx}}(k,R)=k\;E_{\mathrm{elec}}+{\mathrm{kE}}_{\mathrm{amp}}R^{2}=l+{\mu R}^{2}$$

where ‘m’ is the optimal number of cluster-heads and N is the number of sensor nodes in the networks.

**Figure 1.**
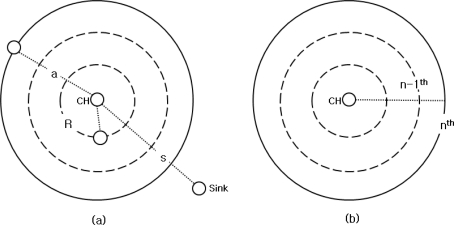
Calculation model for clustering, **(a)** simple cluster modeling, **(b)** multi-hop based communication modeling.

For calculation, we assume the sensor networks are organized as follows: the size of the network is A × A. Sensor nodes are equally distributed over the networks. For simplicity, a cluster-head is located in middle of a local cluster as shown in Figure 1(a). We assume that the distance of the sensor nodes located farthest from the cluster-head is ‘a’ and the radius of a local cluster which is the distance between the cluster-head and a sink, ‘s’. The communication radius of a sensor node is R, (r ≤ R), R is the possible radius of communication within its neighbor nodes and r is the communication radius of sensor nodes [11,12]. Based on these assumptions, we find the energy and size change of a local cluster based on a change in the number of cluster-heads.

The total number of cluster-heads in a network is defined as m, therefore the area of the sensor networks is the same as the total area of local clusters which can be calculated as a^2^π × m = A^2^. From this, we can figure out the radius of a local cluster, ‘a’, as:

(6) $$a=\frac{A}{\sqrt{m\pi }}$$

The number of nodes in a local cluster can be expressed as N/m. As shown in Figure 1(b), a local cluster can be divided into a number of n rings within a radius of nodes, R, for multi-hop communication. The n^th^ ring represents the distance at the farthest nodes, ‘a’. Additionally, it represents hop counts between the cluster-head and the farthest nodes. So, nodes’ hop counts in the n^th^ ring can be described as a/R. We know the average number of sensor nodes with n^th^ hop counts as compared to the area of a local cluster within the area, which is to subtract the area of the (n−1)^th^ ring from the area of the n^th^ ring [14]. Therefore, n^th^_avg_node_, the average number of nodes with n^th^ hop counts is:

(7) $$n_{\mathrm{avg\_node}}^{\mathrm{th}}=\frac{N\left[{\pi a}^{2}-\pi {\left(\mathrm{nR}\right)}^{2}\right]}{{\pi a}^{2}m}$$

The average number of nodes with (n−1)^th^ hop counts, (n−1)^th^_avg_node_ is:

(8) $${\left(n-1\right)}_{\mathrm{avg\_node}}^{\mathrm{th}}=\frac{N\left\{\pi {\left(\mathrm{nR}\right)}^{2}-\pi {\left[\left(n-1\right)R\right]}^{2}\right\}}{{\pi a}^{2}m}$$

When nodes with n^th^ hop counts send data packets to nodes with (n−1)^th^ hop counts, the number of relay packets can be determined by dividing Equation (7) into Equation (8). With n^th^ hop counts in a local cluster, the average number of relay packets, *k_n_Intra_,* is:

(9) $$k_{\mathrm{n\_Intra}}=\frac{N\left[{\pi a}^{2}-\pi {\left(\mathrm{nR}\right)}^{2}\right]/{\pi a}^{2}m}{N\left\{\pi {\left(\mathrm{nR}\right)}^{2}-\pi {\left[\left(n-1\right)R\right]}^{2}\right\}/{\pi a}^{2}m}=\frac{a^{2}-n^{2}R^{2}}{R^{2}\left(2n-1\right)}$$

In an intra-cluster, a node’s energy consumption is divided into two types: transmission energy of own sensing data and relay energy of neighbor’s sensing data. Therefore, the average energy consumption of nodes with n^th^ hop counts, *E*_*mem*_intra_, is to add packet transmission energy, *E_Trans_* to own packet transmission energy, *E_Relay_*, of packet relay energy. This equation can be represented as:

(10) $$\begin{array}{l} E_{{\mathrm{mem}}_{\mathrm{intra}}}=E_{\mathrm{Relay}}\times k_{n_{\mathrm{Intra}}}+E_{\mathrm{Trans}}\\ \;\;\;\;\;\;\;\;\;\;\;\;\;\;\;\;=\left[E_{T_{x}}(k,R)+E_{R_{x}}(k,R)\right]\times k_{\mathrm{n\_Intra}}+E_{T_{x}}(k,R)\\ \;\;\;\;\;\;\;\;\;\;\;\;\;\;\;\;=\left[\left(l+{\mu R}^{2}\right)+l\right]k_{n_{\mathrm{Intra}}}+\left(l+{\mu R}^{2}\right)\\ \;\;\;\;\;\;\;\;\;\;\;\;\;\;\;\;=\left(2l+{\mu R}^{2}\right)\frac{a^{2}-n^{2}R^{2}}{R^{2}\left(2n-1\right)}+\left(l+{\mu R}^{2}\right) \end{array}$$

The average number of packets which a cluster-head receives from member nodes is N/m. The energy consumption of cluster heads for aggregate packets is *E_agg_*. The received packets are sent to a sink node by cluster heads during inter-cluster transmission. At this time, the transmission energy of a cluster head occurs when sending packets to the sink, *E_Trans_*. The average energy consumption of a cluster head, *E_ch_*, can be represented as:

(11) $$E_{\mathrm{ch}}=E_{\mathrm{Agg}}\times \frac{N}{m}+E_{\mathrm{Tran}}=\frac{E_{\mathrm{Agg}}\times N}{m}+\left(l+{\mu R}^{2}\right)$$

Generally, a sink node is located close to the sensor network. The distance between a cluster-head and a sink is from one hop R to n^th^ node’s distance added to one hop R, $R\leq s\leq \sqrt{a}A+R$. Therefore, we can assume that the average distance between a cluster-head and a sink, s, is:

(12) $$s=\frac{\left(\sqrt{2}A+2R\right)}{2}$$

As the number of relay packets is also increased in proportion to s, in inter-clusters, the average number of relay packets, *k_n_Inter_*, is:

(13) $$k_{\mathrm{n\_Inter}}=\frac{\mathrm{sm}}{R}\times \frac{1}{m}=\frac{s}{R}$$

In an inter-cluster, as the CHs can only send the packet to a sink node, we just find the relay packet energy. To achieve this, during inter-cluster transmission, the average energy consumption between a cluster-head and a sink, *E_mem_inter_*, is as follows, according to Equation (13):

(14) $$\begin{array}{l} E_{\mathrm{mem\_inter}}=E_{\mathrm{Relay}}\times k_{\mathrm{n\_Inter}}\\ \;\;\;\;\;\;\;\;\;\;\;\;\;\;\;\;\;\;\;=\left(2l+{\mu R}^{2}\right)\times k_{n_{\mathrm{Inter}}}=\frac{s\left(2l+{\mu R}^{2}\right)}{R} \end{array}$$

Based on the clustering energy equations, we can find the average energy consumption of member nodes, *E_member_*, by using the following added equations:

(15) $$\begin{array}{l} E_{\mathrm{member}}=E_{{\mathrm{mem}}_{\mathrm{Intra}}}+E_{{\mathrm{mem}}_{\mathrm{Inter}}}\\ \;\;\;\;\;\;\;\;\;\;\;\;\;\;\;=\left(2l+{\mu R}^{2}\right)\frac{a^{2}-n^{2}R^{2}}{R^{2}\left(2n-1\right)}+\left(l+\mu R^{2}\right)+\left(2l+\mu R^{2}\right)\frac{s}{R} \end{array}$$

## 4. Performance Analysis and Results

### 4.1. Network Configuration

For network configuration, we assume the following network topology, as described in Table 1.

We set up the size of the networks to be 100 m × 100 m, with a possible node communication radius, R, set at 5 meters. To prevent an isolated node, the number of network nodes is 400. The sensor node’s initial energy is 1 J (Joule) and the data packets of a node are 525 bytes between a cluster-head and member node, and a sink and a cluster-head. As described previously, a sink node is located outside of the sensor networks with the distance between a sink and the networks defined as R. For constant energy, we set up the transmission/receiving energy, *E_elec_*, to be 50 nJ/bit and the amplifier energy, *E_amp_*, to be 10 pJ/bit/m^2^. The aggregation energy per a packet, *E_f_* or *E_agg_*, is 0.21 mJ/bit [4]. After cluster configuration, we assume that the process of transmitting sensing data to a sink is one round. Thus, member nodes send their own sensing data packet to cluster-heads, cluster-heads aggregate and process them, and then the cluster-heads transmit the processed data to a sink over a data path. In these processes, we omit the messages or packets for clustering as negligible.

### 4.2. Performance Results

**Figure 2.**
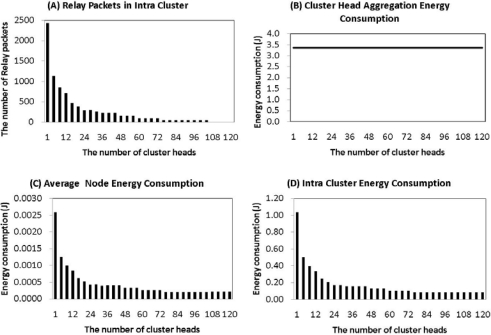
Intra-Cluster. **(A)** relay packets in intra-clusters, **(B)** cluster-head aggregation energy consumption, **(C)** average node energy consumption, **(D)** intra-cluster energy consumption.

In an intra-cluster scenario, if there is a single cluster-head, as illustrated in Figure 2(A), the number of relay packets of member nodes is more than 2,400 per round, based on multi-hop communication. As the number of cluster-heads increases, Figure 2(A) shows that the number of relay packets is decreased.

The reason for this is that the average distance between a cluster-head and member nodes is decreased. In the case of cluster-heads being more than 27% of the networks, a local cluster does not generate the relay packets, as the distance between a cluster-head and member nodes is a < R. On the other hand, the packets which a cluster-head collects from member nodes are fixed, regardless of changing the number of cluster-heads, because the total aggregated data from nodes is always 400, as seen in Figure 2(B). Therefore, the average energy consumption of a node only affects intra-cluster energy consumption, as seen in Figure 2(C). As shown in Figure 2(C,D), the energy consumption of a node is in proportion to the intra-cluster energy consumption.

**Figure 3.**
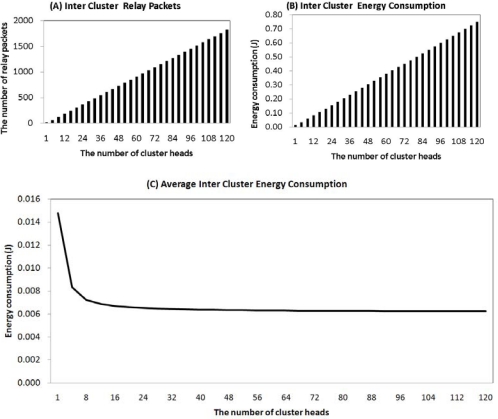
Inter-Cluster. **(A)** Inter-cluster relay packets, **(B)** Inter-cluster energy consumption, **(C)** average inter-cluster energy consumption.

In an inter-cluster, with an increase of cluster-heads, there is an increase of relay packets between a cluster-head and a sink. As illustrated in Figure 3(A), if sensor networks have only one cluster-head, there are 15 relay packets in the inter-cluster. When cluster-heads are more than 30% of the sensor networks they send more than 1,800 relay packets to a sink. An increase in the number of relay packets affects inter-cluster energy consumption, as seen in Figure 3(B). This is the reason most of the energy consumption in an inter-cluster is only affected by the relay packets which cluster-heads transmit to a sink. Even if the inter-cluster energy is increased, the average inter-cluster energy consumption is fixed after 12 cluster-heads, 3% among sensor nodes, as shown in Figure 3(C), because increased cluster-heads distribute the energy loads evenly in the networks.

**Figure 4.**
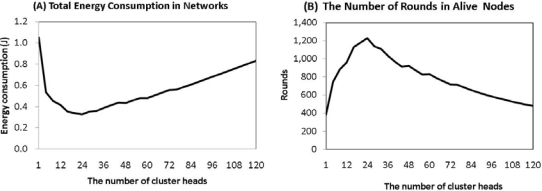
Energy consumption and node alive. **(A)** total energy consumption in the network, **(B)** the number of rounds in alive nodes.

In Figures 2(D) and 3(B), the amount of intra-cluster energy is inversely proportional to the amount of inter-cluster energy. Therefore, it is important to obtain a balance between the two. Using the two energy consumptions, we can find the total energy consumption of sensor networks, as shown in Figure 4(A). In this figure, in the case of a single cluster-head, the total energy consumption of the network is 1.05 J. This value is very large compared to the initial sensor energy. If the number of cluster-heads is 24, or 6%, the total energy consumption is minimal at 0.32 J. If the number of cluster heads number is more than 24, the energy consumption is gradually increased. Figure 4(A) shows that the ratio of cluster-heads should be between 4% and 8% in order to consume the minimum amount of energy. Considering the average energy consumption per round, we can find the number of rounds of a node. As seen in Figure 4(B), in the case of only one cluster-head, a node can be alive below 400 rounds. In the case of 24 cluster-heads however, a node can be alive for more than 1,200 rounds. So, to establish 4% to 8% of cluster-heads among sensor nodes is the most energy efficient method to set up for the optimal number of cluster-heads.

## 5. Conclusions

In previous clustering algorithms for WSNs, they did not consider the practical transmission radius of sensor nodes for communicating with nodes in and out of clusters. All sensor nodes, however, should use the multi-hop method as they have a restricted communication radius. In multi-hop based communication, communication messages or packets in the WSNs are increased in proportion to the distance between nodes. Also, clustering algorithms are affected by the distance. In any clustering algorithm, packet delivery distance is an important issue and is influenced by the number of clusters. All this is related to energy consumption in WSNs. We need to know how many clusters are best in WSNs. Thus we propose the optimal number of cluster-heads based on changing the number of cluster-heads and the associated consumed energy. To prove this, we calculated the energy change in the total network consumption using the number of relay packets in intra-cluster and inter-cluster transmission. We found the change ratio of a cluster-head’s energy, the change ratio of intra-cluster energy, and the change ratio of inter-cluster energy based on a sensor energy model and relay packets by experiments. Therefore, we determined that a change in the number of cluster-heads affects the consumed energy of a sensor network and were thus able to determine the optimal number of cluster-heads a sensor network requires.

## Figures and Tables

**Table 1.** System parameter for network configuration.

**Parameter**	**Value**
Network size	100 × 100 (100 m^2^)
Sensor nodes	400
Radius of sensor nodes	5 m
Data packet	525 bytes
*E_elec_*	50 nJ/bit
*E_amp_*	10 pJ/bit/m^2^
*E_f_*	0.021 mJ
Initial Energy	1 J
